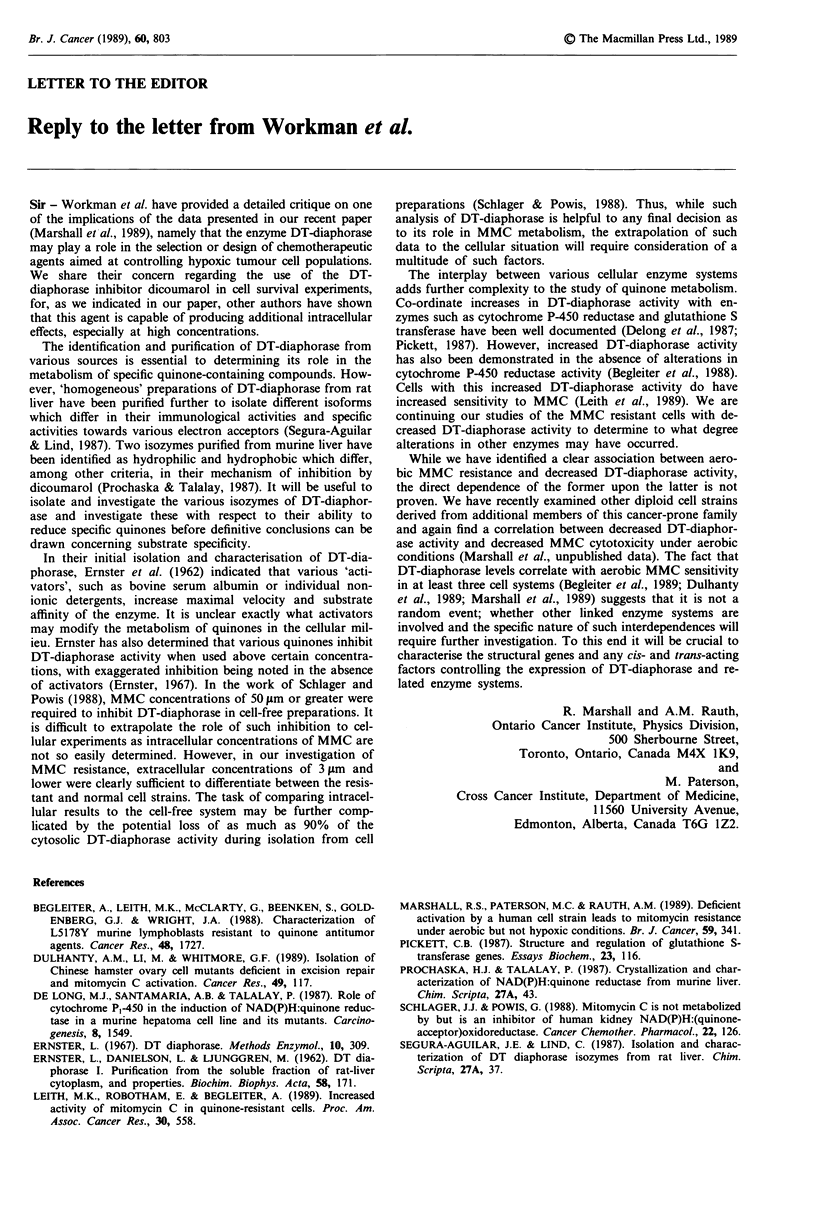# Reply to the letter from Workman et al

**Published:** 1989-11

**Authors:** R. Marshall, A.M. Rauth, M. Peterson


Br. J. Cancer (1989), 60, 803 © The Macmillan Press Ltd., 1989

### LETTER TO THE EDITOR

### Reply to the letter from Workman *et al.*

**Sir** – Workman *et al.* have provided a detailed critique on one of the implications of the data presented in our recent paper (Marshall *et al.*, 1989), namely that the enzyme DT-diaphorase may play a role in the selection or design of chemotherapeutic agents aimed at controlling hypoxic tumour cell populations. We share their concern regarding the use of the DT-diaphorase inhibitor dicoumarol in cell survival experiments, for, as we indicated in our paper, other authors have shown that this agent is capable of producing additional intracellular effects, especially at high concentrations.

The identification and purification of DT-diaphorase from various sources is essential to determining its role in the metabolism of specific quinone-containing compounds. However, 'homogeneous' preparations of DT-diaphorase from rat liver have been purified further to isolate different isoforms which differ in their immunological activities and specific activities towards various electron acceptors (Segura-Aguilar & Lind, 1987). Two isozymes purified from murine liver have been identified as hydrophilic and hydrophobic which differ, among other criteria, in their mechanism of inhibition by dicoumarol (Prochaska & Talalay, 1987). It will be useful to isolate and investigate the various isozymes of DT-diaphorase and investigate these with respect to their ability to reduce specific quinones before definitive conclusions can be drawn concerning substrate specificity.

In their initial isolation and characterisation of DT-diaphorase, Ernster *et al.* (1962) indicated that various 'activators', such as bovine serum albumin or individual non-ionic detergents, increase maximal velocity and substrate affinity of the enzyme. It is unclear exactly what activators may modify the metabolism of quinones in the cellular milieu. Ernster has also determined that various quinones inhibit DT-diaphorase activity when used above certain concentrations, with exaggerated inhibition being noted in the absence of activators (Ernster, 1967). In the work of Schlager and Powis (1988), MMC concentrations of 50 $\mu$m or greater were required to inhibit DT-diaphorase in cell-free preparations. It is difficult to extrapolate the role of such inhibition to cellular experiments as intracellular concentrations of MMC are not so easily determined. However, in our investigation of MMC resistance, extracellular concentrations of 3 $\mu$m and lower were clearly sufficient to differentiate between the resistant and normal cell strains. The task of comparing intracellular results to the cell-free system may be further complicated by the potential loss of as much as 90% of the cytosolic DT-diaphorase activity during isolation from cell preparations (Schlager & Powis, 1988). Thus, while such analysis of DT-diaphorase is helpful to any final decision as to its role in MMC metabolism, the extrapolation of such data to the cellular situation will require consideration of a multitude of such factors.

The interplay between various cellular enzyme systems adds further complexity to the study of quinone metabolism. Co-ordinate increases in DT-diaphorase activity with enzymes such as cytochrome P-450 reductase and glutathione S transferase have been well documented (Delong *et al.*, 1987; Pickett, 1987). However, increased DT-diaphorase activity has also been demonstrated in the absence of alterations in cytochrome P-450 reductase activity (Begleiter *et al.*, 1988). Cells with this increased DT-diaphorase activity do have increased sensitivity to MMC (Leith *et al.*, 1989). We are continuing our studies of the MMC resistant cells with decreased DT-diaphorase activity to determine to what degree alterations in other enzymes may have occurred.

While we have identified a clear association between aerobic MMC resistance and decreased DT-diaphorase activity, the direct dependence of the former upon the latter is not proven. We have recently examined other diploid cell strains derived from additional members of this cancer-prone family and again find a correlation between decreased DT-diaphorase activity and decreased MMC cytotoxicity under aerobic conditions (Marshall *et al.*, unpublished data). The fact that DT-diaphorase levels correlate with aerobic MMC sensitivity in at least three cell systems (Begleiter *et al.*, 1989; Dulhanty *et al.*, 1989; Marshall *et al.*, 1989) suggests that it is not a random event; whether other linked enzyme systems are involved and the specific nature of such interdependences will require further investigation. To this end it will be crucial to characterise the structural genes and any *cis-* and *trans*-acting factors controlling the expression of DT-diaphorase and related enzyme systems.

R. Marshall and A.M. Rauth,
Ontario Cancer Institute, Physics Division,
500 Sherbourne Street,
Toronto, Ontario, Canada M4X 1K9,
and
M. Paterson,
Cross Cancer Institute, Department of Medicine,
11560 University Avenue,
Edmonton, Alberta, Canada T6G 1Z2.

### References

BEGLEITER, A., LEITH, M.K., McCLARTY, G., BEENKEN, S., GOLDENBERG, G.J. & WRIGHT, J.A. (1988). Characterization of L5178Y murine lymphoblasts resistant to quinone antitumor agents. *Cancer Res.*, **48**, 1727.

DULHANTY, A.M., LI, M. & WHITMORE, G.F. (1989). Isolation of Chinese hamster ovary cell mutants deficient in excision repair and mitomycin C activation. *Cancer Res.*, **49**, 117.

DE LONG, M.J., SANTAMARIA, A.B. & TALALAY, P. (1987). Role of cytochrome $P_1$-450 in the induction of NAD(P)H:quinone reductase in a murine hepatoma cell line and its mutants. *Carcinogenesis*, **8**, 1549.

ERNSTER, L. (1967). DT diaphorase. *Methods Enzymol.*, **10**, 309.

ERNSTER, L., DANIELSON, L. & LJUNGGREN, M. (1962). DT diaphorase I. Purification from the soluble fraction of rat-liver cytoplasm, and properties. *Biochim. Biophys. Acta*, **58**, 171.

LEITH, M.K., ROBOTHAM, E. & BEGLEITER, A. (1989). Increased activity of mitomycin C in quinone-resistant cells. *Proc. Am. Assoc. Cancer Res.*, **30**, 558.

MARSHALL, R.S., PATERSON, M.C. & RAUTH, A.M. (1989). Deficient activation by a human cell strain leads to mitomycin resistance under aerobic but not hypoxic conditions. *Br. J. Cancer*, **59**, 341.

PICKETT, C.B. (1987). Structure and regulation of glutathione S-transferase genes. *Essays Biochem.*, **23**, 116.

PROCHASKA, H.J. & TALALAY, P. (1987). Crystallization and characterization of NAD(P)H:quinone reductase from murine liver. *Chim. Scripta*, **27A**, 43.

SCHLAGER, J.J. & POWIS, G. (1988). Mitomycin C is not metabolized by but is an inhibitor of human kidney NAD(P)H:(quinone-acceptor)oxidoreductase. *Cancer Chemother. Pharmacol.*, **22**, 126.

SEGURA-AGUILAR, J.E. & LIND, C. (1987). Isolation and characterization of DT diaphorase isozymes from rat liver. *Chim. Scripta*, **27A**, 37.